# Optimization and Validation of a Method to Determine Enolones and Vanillin Derivatives in Wines—Occurrence in Spanish Red Wines and Mistelles

**DOI:** 10.3390/molecules28104228

**Published:** 2023-05-22

**Authors:** Mónica Bueno, Julián Zapata, Laura Culleré, Ernesto Franco-Luesma, Arancha de-la-Fuente-Blanco, Vicente Ferreira

**Affiliations:** Laboratory for Aroma Analysis and Enology (LAAE), Departament of Analytical Chemistry, Faculty of Sciences, Universidad de Zaragoza, Instituto Agroalimentario de Aragón-IA2 (Universidad de Zaragoza-CITA) Associate Unit to Instituto de las Ciencias de la Vid y el Vino (ICVV) (UR-CSIC-GR), 50009 Zaragoza, Spain

**Keywords:** polar metabolites, enolones, vanillin derivatives, sotolon, furaneol, vanillin, wine, mistelle, odor activity value

## Abstract

Understanding the chemical nature of wine aroma demands accurate quantitative determinations of different odor-active compounds. Quantitative determinations of enolones (maltol, furaneol, homofuraneol, and sotolon) and vanillin derivatives (vanillin, methyl vanillate, ethyl vanillate, and acetovanillone) at low concentrations are complicated due to their high polarity. For this reason, this paper presents an improved and automated version for the accurate measure of these common trace wine polar compounds (enolones and vanillin derivatives). As a result, a faster and more user-friendly method with a reduction of organic solvents and resins was developed and validated. The optimization of some stages of the solid phase extraction (SPE) process, such as washing with an aqueous solution containing 1% NaHCO_3_ at pH 8, led to cleaner extracts and solved interference problems. Due to the polarity of these type of compounds, an optimization of the large volume injection was also carried out. Finally, a programmable temperature vaporization (PTV) quartz glass inlet liner without wool was used. The injector temperature was raised to 300 °C in addition to applying a pressure pulse of 180 kPa for 4 min. Matrix effects were solved by the use of adequate internal standards, such as ethyl maltol and 3′,4′-(methylenedioxy)acetophenone. Method figures of merit were highly satisfactory: good linearity (r^2^ > 0.98), precision (relative standard deviation, RSD < 10%), high recovery (RSD > 89%), and low detection limits (<0.7 μg/L). Enolones and vanillin derivatives are associated with wine aging. For this reason, the methodology was successfully applied to the quantification of these compounds in 16 Spanish red wines and 12 mistelles. Odor activity values (OAV) indicate that furaneol should be considered an aroma impact odorant in red wines and mistelles (OAV > 1) while homofuraneol and sotolon could also produce changes in their aroma perceptions (0.1 < OAV < 1).

## 1. Introduction

Enolones such as maltol, furaneol, homofuraneol, and sotolon, along with vanillin derivatives that comprise vanillin, methyl and ethyl vanillates, and acetovanillone are volatile molecules commonly present in wines [1]. Within each group of compounds, their chemical structures present certain similarity, and this similarity is related to their analogous physicochemical and olfactory properties.

Sotolon is responsible for curry aroma while maltol, furaneol, and homofuraneol have very sweet aromas such as burnt sugar or candy. Sotolon is the molecule studied in this work that has aroused the greatest interest in different types of wines. Sotolon has been described as the key molecule in the characteristic “spicy-like” aroma in fortified wines [2] and correlates with both wine ageing and sugar presence [3,4]. On the other hand, in the case of dry white wines, the presence of sotolon is considered a defect associated with a decrease in the freshness character due to oxidation [5,6,7]. Therefore, sotolon quantification has been widely studied in Port, Madeira or Sherry wines [2,3,4,8,9,10,11] as well as dry white wines [4,6,10,12]. However, few works quantify sotolon or other enolones in red wines [13,14,15] and, to the best of our knowledge, none in mistelles.

Vanillin, acetovanillone, and methyl and ethyl vanillates are also present sweet aromas, but they are responsible for vanilla aromas. Vanillin derivatives can form from different grape precursors. Nevertheless, the quantities thus formed cannot rival the levels released by some types of oak wood during wine aging [16]. Therefore, the quantification of vanillin derivatives is essential in red or white wines aged with oak chips or oak barrels [17,18,19,20].

Little literature exists on odorant compounds in mistelles [21,22,23], but until now, no one has paid special attention to enolones or vanillin derivatives.

Quantitative determinations of enolones and vanillin derivatives at low concentrations are complicated due to their high polarity [1,24]. The first approaches for the analysis of these compounds were based on classical liquid–liquid extractions with dichloromethane followed by analysis on a gas chromatography-mass spectrometer (GC-MS) [2,3,6]. Almost simultaneously, the use of polymeric resins in solid phase extraction (SPE) showed advantages in the extraction concentration of the compounds, a powerful clean-up of the extract as well as a reduction of the dichloromethane employed [17,24,25]. More recently, new methods based on liquid chromatography have been developed trying to avoid gas chromatograpic peak tails due to the high polarity of these compounds [4,12,26]. However, these liquid chromatography approaches were optimized and validated only for the quantification of one compound, sotolon.

This work aims to improve a previous method for the determination of eight highly polar compounds (enolones and vanillin derivatives) by automated SPE followed by GC-MS. The method was applied to the quantification and odor activity values (OAVs) calculated for enolones and vanillin derivatives in Spanish red wines and, to the best of our knowledge, this is the first time that these compounds have been quantified in mistelles.

## 2. Results and Discussion

The main goal of this paper was to improve a previous method for the accurate measurement of interesting trace wine polar compounds by GC-MS. The major changes applied to the previous methodology versions developed by our own research group [15,24] are listed below, and the most interesting are discussed in following sections.

- The first difference is the number of compounds analyzed, as this new methodology quantifies enolones and vanillin derivatives.
- Ethyl maltol and 3′,4′-(methylenedioxy)acetophenone addition to the initial sample. These compounds have similar chemical formulations as the analytes and, therefore, should perfectly imitate their behavior, particularly regarding the intermolecular interactions that they exert towards different matrix components. Furthermore, their addition before starting the extraction process allows the internal standards (IS) to undergo the same analytical process as the analytes, so it can make the signal of the analytes independent of the injected volume, in addition to small matrix differences, thus reducing matrix effects.
- Optimization of some stages of the extraction process such as the first washing step, the resin drying process or the lack of a need to concentrate the extract, with the time savings that this implies. The study of the washing stage deals with obtaining cleaner samples in order to improve the resolution of the chromatographic peaks and decrease the detection (LDs) and quantification limits (LQs). On the other hand, the introduction of drying stages under nitrogen stream instead of under vacuum helps to prevent the oxidation of the analytes.
- Large volume injection, the use of acquisition windows, and acquisition in single ion monitoring (SIM) mode (Table 1) to gain sensitivity.
- Taking into consideration the societal needs towards more efficient, fast, and green analytical methods, an automatization and re-escalation of the extraction process were carried out. This implies a faster and more user-friendly method than in previous versions, with a reduction of organic solvents and resins.

Moreover, the most relevant improvements introduced into the proposed extraction methodology compared to the previous versions have been detailed in Table 2.

**Table 1:** Masses of the ions selected for the determination of the compounds considered in the study and concentrations added in samples in some validation experiments. Acquisition details and retention times of each analyte.

Acquisition Window	Peak Number	Compound	RT (min)	*m*/*z*	Added (μg/L)
10.00–14.00	1	2-octanol (IS1)	9.897	97	
20.70–21.65	2	Maltol	19.004	126	206
21.66–23.10	3	Ethyl maltol (IS2)	20.010	140	
	4	Furaneol	20.684	128, 85	108
23.12–25.49	5	Homofuraneol	22.124	142	131
25.51–28.49	6	Sotolon	25.533	128, 83	209
28.51–31.49	7	3′,4′-(methylenedioxy)acetophenone (IS3)	29.010	149, 164	
31.51–35.99	8	Vanillin	31.630	151, 152	160
	9	Methyl vanillate	32.069	151, 182	126
	10	Ethyl vanillate	32.378	196, 151	145
	11	Acetovanillone	32.493	166, 151	131

IS: internal standard. RT: retention time. Quantitative *m*/*z* ion is written first.

### 2.1. Method Optimization

#### 2.1.1. First Washing Step

**Figure 1:**
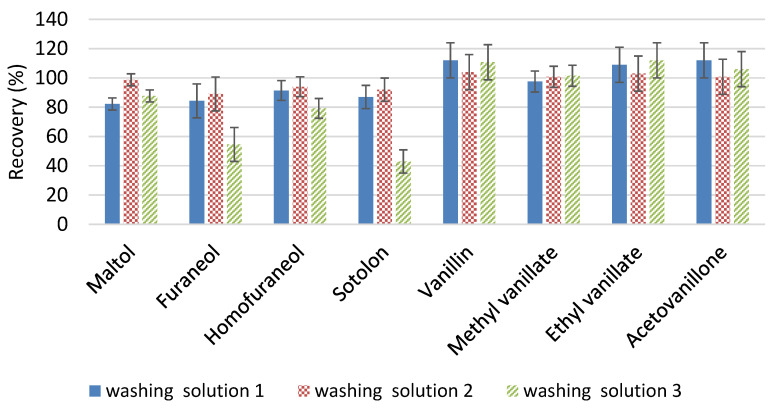
Recovery study with potential washing solvents: (1) water, (2) water solution containing 1% (w/w) of NaHCO_3_ at pH 8, (3) water solution containing 1% (w/w) of NaHCO_3_ at pH 9.

Three different washing solutions were tested: water, a water solution containing 1% (w/w) of NaHCO_3_ at pH 8, and a water solution containing 1% (w/w) of NaHCO_3_ at pH 9. Recovery percentage results are shown in Figure 1. 

The solution at pH 8 improved the recovery of enolones with respect to water, especially in the case of maltol (an increase of 16%). However, at pH 9, significant amounts of homofuraneol were lost, but especially large amounts of furaneol and sotolon (30 and 44%, respectively) disappeared. On the other hand, with respect to vanillin derivatives, the pH of the aqueous washing solution did not show relevance (no statistical differences were found). Therefore, the optimal composition of this first washing stage was established to be an aqueous solution containing 1% NaHCO_3_ at pH 8.

#### 2.1.2. Injection Parameters

**Figure 2:**
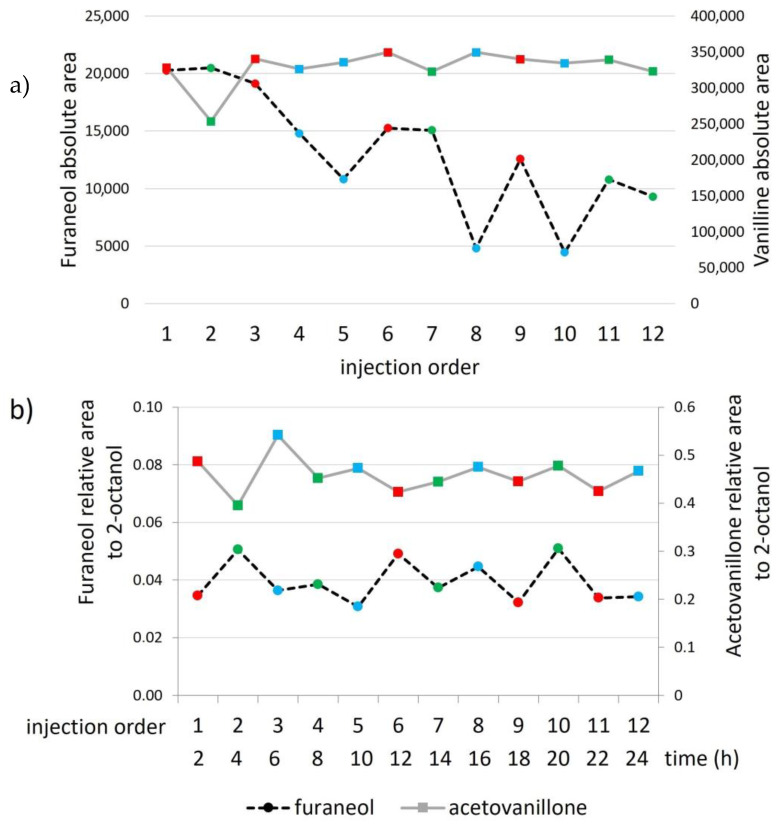
Effect of the sequence and injection order (**a**) before injection optimization, (**b**) after injection optimization. Red: 1st sequence; green: 2nd sequence; blue: 3rd sequence.

**Figure 3:**
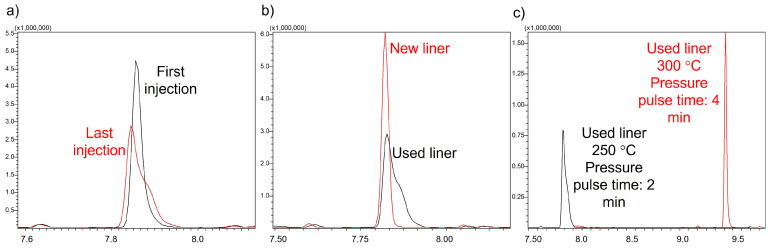
Internal standard 2-octanol peak using a PTV liner filled with deactivated sintered glass. (**a**) First attempt, (**b**) using a new liner, (**c**) after changing the injection conditions.

To study extract stability, twelve samples of the same wine were extracted in three sequences of four samples and randomly injected into the GC-MS. Significant differences (*p* < 0.05) were found for all enolones, while vanillin derivatives seemed to remain stable. Figure 2a shows the results of the absolute areas of furaneol and acetovanillone as an example. It is noteworthy that for enolones, there was both an effect of the injection order and the order of the analysis sequence; in both cases, the signal decreased, indicating stability problems in the extract and/or in the injection. However, after injecting the same extract 10 times, a decrease in the signal of enolones with the injection order continued to be observed, as well as a significant change in the shape of the peak of the internal standard 2-octanol (Figure 3a), which could be indicative of possible adsorption on the insert. The insert used at that time was a programmable temperature vaporization (PTV) liner filled with deactivated sintered glass.

In an attempt to improve the shape of the 2-octanol peak and to evaluate how the effect on the insert activity, the same extract was injected under the same conditions, placing a new insert with the same characteristics. Although the shape of the 2-octanol peak was recovered (Figure 3b) and signal improvements were obtained for all compounds (maltol recovered the most, up to 25%), the signal was not fully recovered compared to the first injections (as an example, 32% of the maltol signal was still missing).

In order to avoid or minimize the liner adsorption phenomenon, the injection conditions were changed. The final injector temperature was raised from 250 °C to 300 °C, and the pulse time was also increased from 2 to 4 min. The 2-octanol signal obtained with these new conditions was satisfactory, and even with the previously used liner, the shape of the 2-octanol peak was recovered (Figure 3c). Nevertheless, despite these changes, when re-injecting the same extract 10 times, it was observed that the adsorption problems had not disappeared, the signal loss of the enolones continued, and the shape of the 2-octanol peak worsened with time. Moreover, the possibility of enolone oxidation in the extract had not yet been excluded.

Theretofore, all previous results seemed to illustrate an insert adsorption effect rather than an extract degradation. However, both effects were studied at the same time. With the aim of minimizing the active points in the filling of the insert, a conventional PTV liner without any type of filling was used. In addition, antioxidant butylated hydroxyanisole (BHA) or butylated hydroxytoluene (BHT) (10 µL 1% w/w solution in dichloromethane) was added to prevent extract oxidation.

The antioxidant BHA interfered with vanillin derivatives; thus, only the same extract with and without BHT was analyzed. It was injected eight times each. The means of the absolute areas of the peaks in each condition were compared using a *t*-test, without finding significant differences in any case (*p* > 0.05). Therefore, oxidation was discarded.

**Figure 4:**
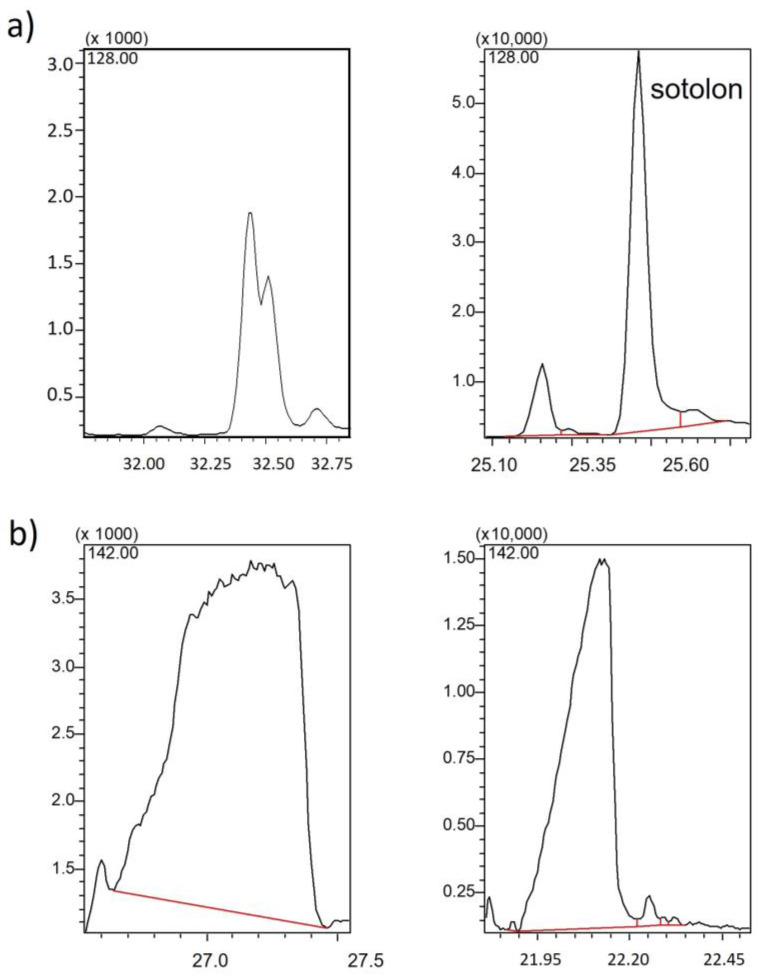
Effect of the oven temperature gradient on (**a**) sotolon and (**b**) homofuraneol. From 180 °C to 240 °C at 2 °C/min (left) or at 20 °C/min (right).

The use of the new insert without filling provided satisfactory results since activity problems were minimized. In exchange for this improvement, a new problem was observed: now the sotolon peak presented an interference that did not exist before, possibly due to the retention of the compounds in the filling of the previous insert. To solve this problem, the speed of the temperature gradient of the oven in the sotolon elution zone was increased from 2 °C/min to 20 °C/min. In this way, the sotolon peak was resolved (Figure 4a), and the shape of the homofuraneol peak was also significantly improved (Figure 4b). Indeed, not only homofuraneol but also its tautomer 5-ethyl-4-hydroxy-2-methyl-3(2H)-furanone could be quantified. These isomers (both with 2 enantiomers) are naturally occurring as a result of their spontaneous racemization due to their keto-enol structures [27]. For this reason, the width of the initial peak of homofuraneol was 1 min, and an increase in the temperature gradient caused the tautomers’ retention times to coincide, providing a peak with better shape.

**Figure 5:**
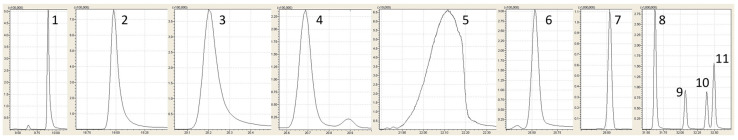
MS ion chromatogram of target compounds obtained with the proposed procedure. Compound identification and *m*/*z* are shown in Table 1.

Finally, the acquisition windows of the chromatographic method (Table 1) were adjusted to be able to acquire the same *m*/*z* for a longer time and thus increase the signal-to-noise ratio (*S*/*N*). The summary of the optimal working conditions is described in Section 3.3.2 (proposed methodology). The final chromatogram is indicated in Figure 5.

#### 2.1.3. Extracts’ Stability

Finally, after finding the optimal injection conditions and solving the sotolon resolution problems, the stability of the eluates was controlled after the first 24 h. As observed in Figure 2b, the slope of the regression lines formed by the 12 samples analyzed in three sequences and injected in random order did not differ significantly from zero (*p* > 0.05), indicating that the extracts from the samples could be left in the tray for at least 24 h.

#### 2.1.4. Automated SPE

As expected, no significant differences (*p* > 0.05) were observed for any analyte based on the average result of each wine when both types of SPEs (semiautomated and automated) were compared using a paired *t*-test.

The reproducibility of each analyte using the semiautomated and automated SPE approaches was assessed through the combined relative standard deviation percentage (RSD%) obtained from the RSD% of the six tested wines. In the case of the semiautomatic SPE, the RSD ranged from 9 to 14%, while the automated SPE obtained values between 4 and 9%, always reaching lower values in the case of the automated SPE, unsurprisingly.

### 2.2. Method Validation

Method quality parameters were evaluated after the optimum conditions were established. Figures of merit for the method sensitivity and precision are shown in Table 3.

Injection repeatability was evaluated due to the polar character of the analytes, which, as previously discussed, could lead to adsorption problems. Repeatability results from 10 consecutive determinations of the same extract were very satisfactory, showing values less than 4% for all compounds.

The reproducibility results aided in the determination of the best internal standard for each analyte. In almost all cases, a lower percentage of RSD was reached for the internal standard with a chemical structure more similar to the analytes (ethyl maltol for enolones and 3′,4′-(methylenedioxy)acetophenone for vanillin derivatives). The worst result was obtained for furaneol, whose reproducibility related to ethyl maltol was 10%. Only in the case of acetovanillone could any of the three standards be used.

Method sensitivity was evaluated in terms of LDs and LQs. The detection limits obtained for all analyzed compounds were lower than or equal to 0.7 μg/L. These values were adequate to evaluate the sensory contribution of these wines’ compounds since they were well below the corresponding threshold values (see Table 3). In general, these LD values are at least half compared to other previously published data for the analysis of enolones [2,3,4,6,24,31] or vanillin derivatives [19,22,32]. Only the LDs of methyl and ethyl vanillate analyzed by the trace wine volatile compounds method of Lopez et al. [25] are very similar (0.49 and 0.17 μg/L, respectively). Exclusively for the analysis of sotolon, Gabrielli et al. [12,26] achieved even lower detection limits. However, it should be noted that the three referenced methods were based on a previous liquid–liquid extraction of 30 mL of wine using not less than 20 mL of dichloromethane, followed by a purification process with resins before the analysis by liquid chromatography. In addition, for the methodologies described in 2015, sotolon could not be quantified in 10% of the dry white wines studied due to the presence of an interfering peak.

To evaluate both the linearity and a preliminary assessment of matrix effects, three calibration experiments were developed by means of standard additions to a young red wine, an aged red wine, and a mistelle (Table 4). For each type of sample, two calibration plots were constructed: one using 2-octanol as IS and the other one using the IS for which the best reproducibility results were obtained (ethyl maltol, IS2, for enolones and 3′,4′-(methylenedioxy)acetophenone, IS3, for vanillin derivatives).

The calibration lines built using 2-octanol as IS were linear (r^2^ > 0.99 except for ethyl vanillate); nevertheless, the statistical study carried out on their slopes showed significant differences in almost all compounds with the exception of vanillin and acetovanillone (Table 4a). This indicates that although 2-octanol was a necessary standard for the injection control of the method, it was not capable of correcting the matrix effect between samples. On the other hand, when ethyl maltol and 3′,4′-(methylenedioxy)acetophenone were used as IS, no significant differences were found between any of the calibration slopes (Table 4b). These results confirmed that an internal standard with a chemical structure similar to that of the analytes added before the extraction process was needed for the correct evaluation of the polar analytes studied in this work.

Moreover, the last column of Table 4b shows that the relative standard deviations of the slopes of each compound assayed were the same order of magnitude as the reproducibility of the method. This result together with the lack of significant differences in the slopes suggests that both enolones and vanillin derivatives were extracted in an equivalent manner in these three samples, and therefore, the average slope can be used to carry out the calibration.

The optimal results presented in Table 4b show that for all analytes, linearity was satisfactory with determination coefficients higher than 0.98. As can be seen, the studied linear ranges spanned more than two orders of magnitude and covered the normal range of occurrence of these compounds in wine [14]. However, it is possible that the method has a greater linear range.

Finally, for the estimation of method accuracy, a recovery study with three other different samples was carried out. As can be seen in Table 5, recoveries were in all cases in the 80–110% range while average recoveries were between 89 and 105%. Furthermore, average recoveries were not significantly different from 100%, as the *t*-test demonstrated. These results confirmed the lack of matrix effects. The standard deviation of the recoveries given in the table provides an estimation of the uncertainty associated with the variability of the matrix. The poorest results were found for furaneol, with a value of 12.

### 2.3. Levels of Enolones and Vanillin Derivatives in Spanish Red Wines and Mitelles

**Figure 6:**
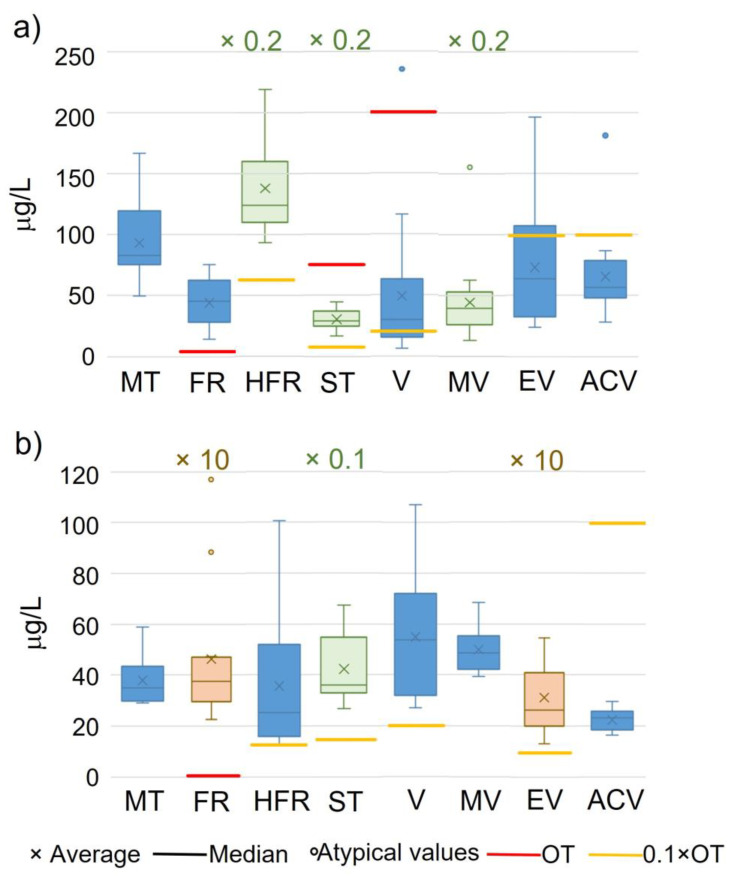
Box plot showing the range of levels of enolones and vanillin derivatives in (**a**) Spanish commercial red wines and (**b**) mistelles. MT: maltol; FR: furaneol; HFR: homofuraneol; ST: sotolon; V: vanillin; MV: methyl vanillate; EV: ethyl vanillate; ACV: acetovanillone. OT: odor threshold. Box plots in different colors indicate different scales.

The optimized and validated method was finally applied to the analysis of enolones and vanillin derivatives in 16 Spanish red wines and 12 mistelles. The results of the analysis summarized in Figure 6 revealed that all samples contained detectable amounts of all of the studied compounds. Even for most of the mistelles, dilution had to be performed to ensure that furaneol and ethyl vanillate could be quantified using the studied linear range.

Wine aroma perception is highly complex, not only due to the large number of odorant compounds present, but also because of the influence of the matrix (which could affect compounds’ volatility) and psychological variables that are involved in wine consumption [33]. A preliminary and simplified approach to investigate the contribution of individual odorants to the aroma of a given food is the use of OAVs, defined as the ratio between the concentration of an odorant in the food and its odor threshold in an appropriate matrix [34]. Therefore, within the 800 odorants found in wine, only a few play noticeable roles in the sensory perception of each wine [35]. Some authors defined the odorants with OAV ≥ 1 as key food odorants (KFOs) [36]; however, lower impact odorants (0.1 ≤ OAV < 1) can produce a profound change in an odor of a mixture [37], leading to competitive, cooperative, destructive or creative interactions [38].

Figure 6 shows the odor thresholds and their values ten times lower (red and yellow lines, respectively). These lines divide the sensory impact space of each analyte. A compound with its occurrence range above the odor threshold means that it has an OAV > 1 and therefore is a KFO. When the occurrence range of a compound is between both lines (red and yellow), this implies that this compound could exert changes in a particular sensory perception due to different interactions with other compounds. Finally, if no line is visible in the figure, this indicates that the range of occurrence is below the yellow line, and consequently it can be assumed that this compound has no sensory relevance (OAV < 0.1).

For all analytes, OAVs were estimated by dividing the concentration level of each compound by its corresponding odor threshold (Table 3), as stated above.

This study, although performed on a limited number of Spanish red wines and mistelles, indicates that furaneol should be considered an aroma impact odorant because all samples exhibited OAV > 1. Homofuraneol and sotolon do not occur at concentrations higher than their odor threshold but they present OAVs > 0.1. It has been demonstrated that low level additions of sotolon could strongly impact the overall sensorial perception of a wine [39], while homofuraneol may contribute to the red fruit notes in red wines [40] in combination with furaneol and maltol since they are part of the same aroma vector [37]. The same occurs with vanillin and ethyl vanillate concentrations in mistelles. Their OAVs are always higher than 0.1 but less than 1, which implies a possible effect on the vanilla aroma vector along with methyl vanillate and acetovanillone [37]. This vanilla vector is directly implicated in sweet and spicy notes [41]. These are matters that need further chemosensory ratification experiments.

## 3. Materials and Methods

### 3.1. Reagents, Standards, Materials, and Samples

#### 3.1.1. Solvents and Reagents

Dichloromethane, methanol, and pentane (gas chromatography quality) were purchased from Merk (Darmstadt, Germany). Ethanol and sodium hydrogen carbonate (NaHCO_3_ 99.7%) were obtained from Panreac (Barcelona, Spain). Water was purified in a Milli-Q system from Millipore (Bedford, Germany). Butylated hydroxytoluene (BHT) and butylated hydroxyanisole (BHA) were obtained from Sigma–Aldrich (Madrid, Spain).

#### 3.1.2. Solid Phase Extraction (SPE)

LiChrolut EN^®^ resins (styrene/divinylbenzene copolymer) and 1 mL internal volume polypropylene cartridges were supplied by Merk (Darmstadt, Germany). These resins were selected due to their demonstrated highest retention for neutral molecules of different polarities [42]. Semiautomated SPE was carried out with a VAC ELUT 20 station system from Varian (Walnut Creek, CA, USA). Automated SPE was carried out in a GX-274 Liquid Handler from Gilson (Middleton, WI, USA).

#### 3.1.3. Chemical Standards

The chemical standards (four enolones and four vanillin derivatives) and the internal standards were supplied by Sigma–Aldrich, with the exception of maltol and furaneol, which were purchased from Fluka (Madrid, Spain). Chemical standards: maltol (3-hydroxy-2-methyl-4H-pyran-4-one) > 98%, furaneol (4-hydroxy-2,5-dimethyl-3(2H)-furanone) >99%, homofuraneol (2-ethyl-4-hydroxy-5-methyl-3(2H)-furanone) >97%, sotolon (3-hydroxy-4,5-dimethyl-2(5H)-furanone) > 97%, vanillin (4-hydroxy-3-methoxybenzaldehyde) ≥99%, methyl vanillate (methyl 4-hydroxy-3-methoxybenzoate) ≥99%, ethyl vanillate (ethyl 4-hydroxy-3-methoxybenzoate) ≥ 99% and acetovanillone (4′-hydroxy-3′-methoxyacetophenone) ≥ 98%. Internal standards (IS): 2-octanol 98%, ethyl maltol (2-ethyl-3-hydroxy-4H-pyran-4-one) ≥ 99%, and 3′,4′-(methylenedioxy)acetophenone 98%.

#### 3.1.4. Samples

For the method development and validation, six different samples were used: two young red wines (YRW1, YRW2) from Somontano and Campo de Borja (Spain), two aged red wines (ARW1, ARW2) from La Rioja and Ribera de Duero (Spain), and two mistelles (MTL1, MTL2) from Cariñena (Spain).

The proposed method was further applied to the analysis of enolones and vanillin derivatives in sixteen red dry wines and twelve mistelles from different Spanish production areas. All samples were commercially available and were made with eight grape varieties and belonged to six different vintages. Ethanol concentration ranged from 12.5 to 14.2% (v/v) for wines and from 12 to 18% (v/v) for mistelles. Oak aging of wines ranged from 0 to 18 months. All measurements were conducted in duplicate.

### 3.2. Method Optimization

Enolones and vanillin were initially extracted and analyzed following the variations carried out by San Juan et al. [15] based on the method proposed by Ferreira et al. [24].

#### 3.2.1. First Washing-Up Step Optimization

The effect of the composition of this first washing step on the recovery of the 4 enolones and 4 vanillin derivatives was studied. The SPE beds were washed with water and water solutions containing 1% (w/w) of NaHCO_3_ at pH 8 or pH 9. The changes in relative areas to 2-octanol of each analyte obtained by adding ≈10 µg/L of each of them to a wine were compared with the changes in the relative areas resulting from adding ≈50 µg/L of each analyte directly to an extract. For this experiment, the method began with 3 mL of wine, and finally 600 µL of extract was obtained, so analytes were concentrated by a factor of 5. The experiment was carried out in duplicate.

#### 3.2.2. Injection and GC-MS Parameter Optimization

For the optimization of the chromatographic conditions, different series of 10 injections of a red wine extract were analyzed repeatedly. Different liners recommended for the large volume injection were tested, such as a sintered glass liner and quartz glass inlet liner without wool, both from Shimadzu (Kyoto, Japan). Furthermore, the injection program was optimized, and finally the program of the chromatographic oven was readjusted, as well as the acquisition windows.

#### 3.2.3. Extract Stability

To verify the stability of extracts, twelve samples of the same wine were analyzed using automated SPE in 3 sequences of 4 samples per sequence and were randomly injected into the GC-MS for a period of 24 h. A *t*-test compared whether the slope of the regression line for each compound differed significantly from 0.

#### 3.2.4. Automated SPE

A comparison between semiautomated and automated SPE was carried out by evaluating the relative areas to 2-octanol of six wines. Each wine was analyzed in triplicate on the same day.

### 3.3. New Proposed Method

#### 3.3.1. Extraction Procedure

The automated SPE of enolones and vanillin derivatives was performed using the automated GX-274 Liquid Handler. In the new proposed method, 3 mL of wine containing 0.9 g of ammonium sulphate was diluted to 6 mL with milli-Q water. Before the extraction process, 60 μL of an internal standard solution containing 3′,4′-(methylenedioxy)acetophenone and ethylmaltol (20 mg/L in ethanol) was added to the diluted wine. This solution (3 mL) was loaded in a 50 mg LiChrolut EN^®^ cartridge previously conditioned with 1 mL of dichloromethane, 1 mL of methanol, and 1 mL of a 12% ethanol (v/v) aqueous solution. After this, the bed was washed with 2 mL of NaHCO_3_ 1% (v/v) aqueous solution (pH = 8) and dried under nitrogen stream. Another washing step was carried out with 2 mL of a 5% of dichloromethane in pentane solution (v/v), and finally, cartridges were dried under nitrogen stream before the elution step. The analytes were eluted with 600 μL of dichloromethane with 5% (v/v) methanol drop by drop. The recovered solution was spiked with 20 μL of the internal standard solution containing 2-octanol (65 mg/L in dichloromethane). Eluates were analyzed in the GC-MS system.

#### 3.3.2. GC-MS Conditions

GC-MS analysis was carried out on a Shimadzu QP-2010 gas chromatograph with a quadrupole mass spectrometric detection system. The injection was carried out in the large volume mode typical of a PTV injector using a quartz glass inlet liner without wool for PTV injection. The initial injector temperature was 65 °C for 0.20 min, which was increased to 300 °C at 400 °C/min and held at that temperature for 15 min. After this, a rate of −400 °C/min was applied to return to the initial temperature (65 °C). The injection was splitless, and after 4.20 min, the split valve was opened. A pressure pulse of 180 kPa was applied during 4 min (the column flow during this period was 3.30 mL/min). In total, 5 microliters was injected. The carrier gas was He at a constant linear velocity of 40 cm/s (≈1.22 mL/min flow rate). The column was a DB-WAXETR capillary column from J&W (Folsom, CA, USA), 30 m × 0.25 mm i.d., with 0.25 μm film thickness, preceded by a silica precolumn from Supelco (Bellefonte, PA, USA) 3 m × 0.25 mm i.d. The chromatographic oven was held at 50 °C for 4.20 min, then raised to 180 °C at 10 °C/min and finally to 240 °C at 20 °C/min, remaining at that temperature for 15 min. The ion source was operated in electron impact (EI) mode. The temperature of the ion source was 220 °C, and the transfer line was kept at 240 °C. The mass analyzer was operated in SIM mode. The solvent cut window was 9.8 min. Quantitative data were obtained by interpolation using the corresponding calibration graphs.

### 3.4. Method Validation

Injection repeatability was determined by evaluating the signal obtained in 10 determinations of the same extract obtained from a red wine spiked with analytes at an approximate concentration of 10 μg/L.

Reproducibility was evaluated by the analysis of two wines and one mistelle spiked or not with known concentrations of all analytes (see Table 1). Each sample determination was carried out in triplicate; each replicate was analyzed on a different day. Method precision was studied using relative areas to 2-octanol, ethyl maltol, and 3′,4′-(methylenedioxy)acetophenone.

Linearity was calculated by standard addition of two red wines and one mistelle using 5 levels of concentrations and two replicates at each level. Calibration graphs using relative areas of different internal standards were calculated. Calibration graphs slopes were compared by analysis of variance (ANOVA). The value of *p* ≤ 0.05 was considered statistically significant.

The evaluation of matrix effects was estimated by a recovery study carried out on two red wines and one mistelle spiked or not with analytes at the levels indicated in Table 1. Each sample was analyzed by the proposed method in triplicate.

Detection limits were defined as the concentration that yielded a *S*/*N* of 3. Quantification limits were calculated as the concentration that resulted in a peak height ten times *S*/*N*.

## 4. Conclusions

This work presents a quick, user-friendly, accurate, and sensitive procedure that allows the determination of highly polar compounds in wine, such as enolones and vanillin derivatives. The proposed method is based on a solid phase extraction and only requires 3 mL of wine. The obtained extract is directly injected (5 µL) into a GC-MS. This procedure has been applied to quantify the eight analytes of interest in 16 red wine samples from different Spanish regions and 12 mistelles. Results revealed that furaneol should be studied as a key red wine and mistelle odorant (OAV > 1), while homofuraneol and sotolon (0.1 < OAV < 1) could also collaborate in changes of aroma perception of these products through aromatic interactions of different types.

## Figures and Tables

**Table 2:** Improvements introduced over time in the extraction of trace wine polar compounds.

Parameters	Ferreira et al., 2003 [24]	San Juan et al., 2011 [15]	Proposed Methodology
Adsorbent mass	800 mg	200 mg	50 mg
Conditioning step	8 mL MeOH 8 mL water/EtOH 13% v/v	6 mL MeOH 6 mL water/EtOH 13% v/v	1 mL DCM 1 mL MeOH 1 mL water/EtOH 13% v/v
Wine volume	50 mL (+7.5 g (NH_4_)_2_ SO_4_)	3 mL wine + 3 mL water (+0.9 g (NH_4_)_2_ SO_4_)	3 mL wine + 3 mL water (+0.9 g (NH_4_)_2_ SO_4_)
Addition of IS to the sample	NO	NO	YES
1st washing step	5 mL water	1.5 mL water	3 mL water containing 1% NaHCO_3_ pH 8
1st drying step	by applying vacuum 30 min	by applying vacuum 30 min	under nitrogen stream 30 min
2nd washing step	PEN:DCM (95:5, v/v) 15 mL	PEN:DCM (95:5, v/v) 6 mL	PEN:DCM (95:5, v/v) 2 mL
2nd drying step	NO	NO	under nitrogen stream 10 min
Elution	6 mL DCM (+2-octanol)	1.5 mL DCM:MeOH (95:5, v/v) (+2-octanol)	600 µL DCM:MeOH (95:5, v/v) (+2-octanol)
Concentration	up to 0.1 mL	up to 0.5 mL	NO
SPE system	S.A. system	S.A. system	A. system

IS: internal standard; SPE: solid phase extraction; MeOH: methanol; EtOH: ethanol; PEN: pentane; DCM: dichloromethane; S.A.: semiautomated; A.: automated.

**Table 3:** Method sensitivity and precision parameters.

	Repeatability RSD (%) ^a^	Reproducibility RSD (%) ^b^			LD (μg/L)	LQ (μg/L)	OT (μg/L)
		IS1	IS2	IS3			
Maltol	2.79	4.11	2.77	7.74	0.48	1.58	5000 [9]
Furaneol	3.79	11.6	10.3	16.6	0.27	0.89	5 [28]
Homofuraneol	2.00	6.78	5.47	12.3	0.69	2.30	125 [28]
Sotolon	3.02	7.94	5.02	9.88	0.42	1.41	15 [29]
Vanillin	3.19	8.60	7.88	7.30	0.16	0.52	200 [30]
Methyl vanillate	3.26	7.18	5.91	4.11	0.52	1.74	3000 [25]
Ethyl vanillate	3.02	8.68	7.72	3.85	0.18	0.61	990 [25]
Acetovanillone	2.27	6.01	5.08	5.87	0.34	1.13	1000 [25]

^a^: Signal evaluation of 10 determinations of the same extract. ^b^: Relative area evaluation of 3 determinations carried out on different days for 3 different samples. LD: detection limit. LQ: quantification limit. OT: odor thresholds calculated in synthetic wine or white wine. IS1: 2-octano. IS2: ethyl maltol. IS3: 3′,4′-(methylenedioxy)acetophenone.

**Table 4:** Method linearity. (**a**) Using 2-octanol (IS1) or (**b**) ethyl maltol (IS2) for enolones and 3′,4′-(methylenedioxy)acetophenone (IS3) for vanillin derivatives as internal standards. Slope and standard deviation (s) values are presented ×10^−3^.

**(a)**														
		**Linearity** **Range**	**YRW1**			**ARW1**			**MTL1**			***p***	**Average Slope**	**RSD (%)**
		**(μg/L)**	**Slope ^a^**	**s**	**r^2^**	**Slope^a^**	**s**	**r^2^**	**Slope ^a^**	**s**	**r^2^**			
Related to IS1	MT	0.48–512	4.36	0.06	0.9994	5.43	0.27	0.9950	4.80	0.17	0.9995	0.001	4.87	11.1
	FR	0.27–267	3.10	0.11	0.9965	3.51	0.09	0.9988	3.30	0.10	0.9975	0.006	3.30	6.16
	HFR	0.69–325	5.20	0.04	0.9998	5.22	0.10	0.9992	4.80	0.07	0.9990	0.001	5.08	4.72
	ST	0.42–517	3.19	0.18	0.9906	3.50	0.04	0.9998	3.45	0.11	0.9952	0.044	3.38	4.91
	V	0.16–395	12.6	0.23	0.9993	12.5	0.46	0.9973	12.0	0.35	0.9958	0.164	12.4	2.64
	MV	0.52–313	23.2	0.04	1.0000	17.4	0.33	0.9993	18.0	0.19	0.9990	<0.001	19.5	16.3
	EV	0.18–359	6.52	0.91	0.9624	4.75	0.54	0.9748	4.50	0.73	0.9824	0.030	5.26	21.0
	ACV	0.34–325	9.55	0.03	1.0000	9.59	0.47	0.9929	9.20	0.05	0.9931	0.243	9.44	2.24
**(b)**														
		**Linearity** **Range**	**YRW1**			**ARW1**			**MTL1**			***p***	**Average Slope**	**RSD (%)**
		**(μg/L)**	**Slope ^a^**	**s**	**r^2^**	**Slope ^a^**	**s**	**r^2^**	**Slope ^a^**	**s**	**r^2^**			
Related to IS2	MT	0.48–512	2.49	0.04	0.9993	2.43	0.08	0.9979	2.46	0.20	0.9995	0.873	2.46	1.09
	FR	0.27–267	1.76	0.06	0.9961	1.64	0.10	0.9928	1.70	0.10	0.9975	0.300	1.70	3.63
	HFR	0.69–325	2.94	0.18	0.9997	2.45	0.20	0.9988	2.70	0.29	0.9990	0.097	2.70	9.10
	ST	0.42–517	1.80	0.10	0.9904	1.66	0.07	0.9964	1.75	0.13	0.9952	0.054	1.74	4.26
Related to IS3	V	0.16–395	5.85	0.31	0.9963	6.54	0.47	0.9899	6.21	0.37	0.9958	0.174	6.20	5.53
	MV	0.52–313	7.58	0.19	0.9982	7.18	0.11	0.9996	7.32	0.24	0.9990	0.092	7.36	2.77
	EV	0.18–359	2.27	0.19	0.9861	2.21	0.11	0.9953	2.23	0.75	0.9824	0.988	2.24	1.27
	ACV	0.34–325	4.06	0.15	0.9959	4.28	0.03	0.9999	4.08	0.33	0.9931	0.437	4.14	2.88

YRW: Young red wine; ARW: aged red wine; MTL: mistelle. ^a^: Five levels of concentrations and two replicates at each level. r^2^: coefficients of determination. MT: maltol; FR: furaneol; HFR: homofuraneol; ST: sotolon; V: vanillin; MV: methyl vanillate; EV: ethyl vanillate; ACV: acetovanillone.

**Table 5:** Recoveries and average recoveries with their standard deviation obtained employing the average slope of the three linearity plots using the optimum internal standard and statistical test for checking matrix effects.

	YRW2	ARW2	MTL2	%R Mean	s	t^a^_100_	*p*
Maltol	99	98	102	100	1.96	0.13	0.91
Furaneol	82	103	83	89	12.0	0.51	0.66
Homofuraneol	103	93	92	96	6.08	0.40	0.73
Sotolon	91	92	102	95	6.05	0.47	0.69
Vanillin	108	108	95	104	7.27	0.28	0.80
Methyl vanillate	99	105	98	101	3.60	0.13	0.91
Ethyl vanillate	98	108	107	105	5.30	0.50	0.67
Acetovanillone	103	92	106	101	7.18	0.05	0.97

YRW: Young red wine; ARW: aged red wine; MTL: mistelle; R% mean: average recovery; ^a^ t experimental value (95% significance) for the comparison of the average percentage for recovery versus 100%.

## Data Availability

Data are contained within the article.

## Sample Availability

Samples of the compounds are not available from the authors.
